# Supplementary material for: Are carbon emissions trading and green financial instruments synergistic? -Comprehensive quantitative research based on content analysis
Source: PLoS One. 2024 Mar 7;19(3):e0298601. doi: 10.1371/journal.pone.0298601 (PMC10919601; doi:10.1371/journal.pone.0298601)
Supplement: S1 Data — (ZIP) [file pone.0298601.s001.zip › Data/地级市Z_F工作报告.zip!/║■▒▒/╩«╤▀╩╨/└┤╫╘╣1⁄2╓┌║┼í╛┬φ┐╦╩2╛▌═°í┐_2016.docx]

各位代表：

　　现在，我代表市人民政府向大会作工作报告，请予审议，并请各位政协委员和其他列席人员提出意见。

　　一、“十二五”时期经济社会发展回顾

　　“十二五”是我市面临挑战众多、压力巨大的五年，更是砥砺奋进、硕果累累的五年。五年来，市委团结带领全市人民着眼于生态文明建设和转型跨越发展走在全省前列，全面推进区域性中心城市建设，圆满完成了“十二五”规划目标，城乡面貌发生了深刻变化，开创了改革发展的新局面。

　　——这五年，经济实力跃上新高。2015年全市地区生产总值达1300亿元，年均增长9.2%；地方公共预算收入93亿元，年均增长12.6%，分别是2010年的1.75倍、2.12倍。新增规上、限上企业983家，外贸出口企业570家，外资企业48家。市场主体达26.6万户，比“十一五”末增长1.6倍。十堰经济技术开发区晋升为国家级。

　　——这五年，人民生活明显改善。城乡居民收入大幅提升，城镇、农村居民人均可支配收入分别达24357元和7891元，比2010年增长92.5%和125.5%。新增就业22.16万人，基本建成覆盖城乡的社保体系，低保补助水平增加1.5倍，实现应保尽保。新增保障性住房14.3万套，改造农村危房6.2万户、老旧小区2.7万户。城乡客运一体化，实现“村村通客车”。

　　——这五年，生态建设成效显著。荣获国家卫生城市、全国最佳生态保护城市、省级森林城市、环保模范城市等称号。建成国家和省级自然保护区、森林公园、湿地公园30个。万元生产总值综合能耗连续3年降幅居全省第一。丹江口水库水质达到国家地表水Ⅱ类标准，全市集中式饮用水源地水质达标率100%，入选首批“中国好水”水源地。空气质量在全省保持领先，城区优良天数达80%以上。森林和林地面积、森林蓄积量全省第一，森林覆盖率达63%。

　　——这五年，改革开放全面深化。重点领域和关键环节改革富有成效，政府职能加快转变，市级行政审批项目消减71%，行政权力、行政服务事项削减27%，成为全省审批最少的城市之一。科技、教育、文化、医疗卫生改革持续推进，生态文明体制机制、农村经营管理体制、多层次资本市场建设都取得重大突破。对外开放水平明显提高，共引进项目1001个、到位资金1409亿元，年均增长30%；外贸出口23.1亿美元，年均增长20.1%；利用外资8.1亿美元，年均增长27%。

　　——这五年，一批大事要事办好办成。郧县撤县设区，城市发展空间大幅拓展，城区由山城向山水城迈进，区域性中心城市框架基本显现；18.2万移民任务胜利完成，南水北调中线工程顺利通水；东风与沃尔沃战略合作成功，东风新特商成立运营；武当大兴600年、全国村长论坛等重大活动圆满举办；全国生态文明先行示范区和地下综合管廊试点城市申创成功；武当山机场即将通航，四条高速公路和六县市绕城一级路建成通车，汉十高铁、京能热电、夹河关水电站全面开工，潘口、小漩、龙背湾、三里坪、白沙水电站等一批项目投产，长期制约十堰发展的空间、交通、能源等瓶颈逐步打破。

　　五年来，我们主要做了以下工作：

　　（一）着力推进转型升级，保持经济持续健康发展

　　一是做强做大汽车主导产业。东风商用车重卡新工厂顺利投产，东风动力总成、装备、零部件等五大园区有序推进，汽车产销规模不断扩大，专用车产量增幅达65.2%，汽车工业产值突破1200亿元。二是加快发展地方特色产业。水电开发、农产品加工、生物医药、食品饮料等产业迅速壮大，占全市工业38.5%，生物医药等6个产业成为省级重点成长型产业集群。三是突破性发展现代服务业。去年接待国内外游客突破4000万人次，实现旅游总收入300亿元，分别增长16%、23%，创建中国优秀旅游城市2个、国家级旅游度假区1个，景区总数全省第一，成为全国百强旅游城市。四大物流集聚区加快推进，培育亿元商贸流通企业19家，发展电商企业6000多家，年交易额突破120亿元。健康养老业快速成长，建成福利院72所、养老机构106所。四是千方百计扩投资促消费。全社会固定资产投资累计完成4540亿元，年均增长27.2%，社会消费品零售总额年均增长15.9%，分别是“十一五”时期的4倍、2.1倍。

　　（二）着力强化“三农”工作，巩固和加强农业基础

　　2015年实现农业总产值286.8亿元，年均增长5.6%；农产品加工产值350亿元，年均增长45.6%，分别是2010年的2.2倍、2.7倍。实施“四个百万”工程，特色基地达550万亩，山羊出栏达116万只。新增农产品加工园区6个、农业标准化示范区24个、市以上龙头企业54家、农民专业合作社579个、家庭农场175个、国家地理标志商标12件、“三品一标”产品200个；除险加固水库365座，解决安全饮水130万人；改造和新修县乡村道7146公里，改造“低电压”居民14.2万户；整治土地60.1万亩，新增耕地9.3万亩。

　　（三）着力深化改革开放，增强经济发展新动能

　　一是大力优化发展环境。建立“四个清单”制度，全面推行“三集中、三到位”改革，推进重点项目并联审批，提高审批效率。扎实开展中介机构治理，推进先照后证、三证合一等商事登记改革。积极推进农村土地制度、产权制度、经营体制、社会化服务体制改革，涉农资金整合形成“郧阳模式”。深化财税体制改革，改进预算分配机制，扩大营改增范围，创新财政投入方式，设立创业投资引导基金。二是深入实施创新驱动和质量兴市战略。争创湖北名牌100个，国家汽车零部件质检中心基本建成，武汉国际光谷产业基地十堰产业园获得批准。国家商用车零部件产业集群创新示范区创建取得进展，国家知识产权示范城市通过验收，连续三年荣获全国科技进步先进市称号。三是着力构建开放型经济体系。商检局建成运行。举办了饮水思源光彩十堰行、中外知名企业十堰行等重大主题招商活动，参与中博会、楚商大会、鄂港澳粤、华创会等经贸交流活动。加强对口协作，与北京市对接项目99个，引进项目55个、到资165.7亿元。对口协作产业投资引导基金正式组建，十堰中关村科技成果产业化基地挂牌成立。

　　（四）着力统筹城乡发展，加快构建“一核多支点”格局

　　编制土地利用、城市总规，控制性详规和生态滨江新区等市县乡村规划1162项，实现城乡规划全覆盖。新建发展大道等城市干道30余条，维修市政道路190余条，改造背街小巷和市政道路微循环260余条，建设污水收集管网1900公里，建成西气东输二线十堰支干线，改造城中村54个，天然气入户18万户，城市功能日臻完善。开发利用低丘缓坡，新增城镇建设用地8万亩，茅箭、张湾成为全国国土资源节约集约利用模范区。建成四方山生态公园，顺利通过第三次国家园林城市评审。城镇化率达到52.4%。“两带”建设纵深推进，以县城、中心镇、特色镇和农村新型社区为核心的四级城镇体系基本建立，基础设施和公共服务明显改善。明确发展定位，强化政策支持，强力推进县域经济发展，县域生产总值、规模以上工业增加值分别比2010年增长1倍、1.2倍。

　　（五）着力增进民生福祉，切实保障和改善民生

　　扎实推进“四双”精准扶贫，精准识别贫困村456个、贫困户26.1万户、84.68万人，1.4万名干部驻村帮扶，10.2万名干部结对帮扶13.6万贫困户，累计减贫50万人，扶贫工作受到省委省政府高度赞誉。全面发展社会事业，新（改、扩）建中小学408所、幼儿园290所，新增校舍57万平方米，学前教育三年毛入园率达87%，初中三年巩固率达99%，高中阶段毛入学率达95%，90%县市区通过义务教育基本均衡省级验收。加快现代公共文化服务体系建设，部分公共文化和体育场馆免费开放，社区文化活动室实现全覆盖，参加省第十四届运动会取得佳绩，广播电视综合覆盖率达98.4%。县级公立医院改革实现全覆盖，每千人拥有病床数7.46张，居全省市州前列，区域医疗中心初步形成。继续稳定低生育水平，调整完善生育政策，卫生计生整体水平得到提升。

　　（六）着力建设生态文明，实现保护和发展同步推进

　　坚持“外修生态、内修人文”，扎实推进五城联创。《丹江口库区及上游水污染防治和水土保持“十二五”规划》任务完成，郧阳区纳入国家级生态保护与建设示范区、全国石漠化综合治理试点和全国生态循环农业示范区，郧西县、竹溪县、郧阳区纳入全省水生态文明城市试点县。深入开展清水行动、“向三大污染宣战”等专项活动，五河治理成为全国治污典范，取缔“十小”及污染企业435家。推进绿满十堰，植树造林159万亩。开展农村环境连片综合整治，创建国家和省级生态乡镇（村）101个。淘汰水泥、炼钢、电解铝等落后产能231万吨。推广污水垃圾处理第三方运营机制，污水处理水平居全国前列。

　　（七）着力加强民主法治和精神文明建设，确保社会大局和谐稳定

　　一是强力推进依法治市。自觉接受人大法律监督和政协民主监督，及时向人大报告工作、向政协通报情况，认真办理人大议案，办理人大代表建议797件，办结率100%，满意和基本满意率99.7%；办理代表走访选民建议311件，办结率100%，满意和基本满意率100%；办理政协提案1663件，办结率100%，满意和基本满意率99.3%。严格落实党风廉政责任制，认真开展专项治理，狠抓作风建设。推进审计工作“全覆盖”。先后荣获“全国首批法治城市创建先进单位”、“全国‘六五’普法中期先进城市”称号。顺利完成两届村委会换届，被评为全国和谐社区建设示范城区。国防动员、拥军优属等工作迈上新台阶，可望蝉联全国双拥模范城“八连冠”和全省双拥模范城“九连冠”。二是深入开展文明创建。实现全国文明城市提名城市“两连冠”和省级文明城市“七连冠”，7家单位和9个村镇荣获全国文明单位、文明村镇称号，157个单位被命名为省级文明单位，50人当选湖北好人，2人当选湖北道德模范，18人荣登中国好人榜。“十星”创建实现全覆盖。三是全面建设和谐十堰。建立健全立体化社会治安防控体系，群众安全感测评和治安满意度连续多年位居全省第一方阵。推行阳光信访，信访形势持续好转。落实安全生产责任，安全生产形势稳定向好，连续5年全省考核优秀。实施食品放心工程，保证食品药品安全。

　　全市民族宗教、妇女儿童、外事侨务、对台、气象、档案、地震、人防、残联、老龄、统计、社科、地方志等各方面工作都取得了新的成绩。

　　各位代表，刚刚过去的2015年，我们紧紧围绕市委确定的六大目标，主动适应经济新常态，积极应对下行压力，尽管主导产业和骨干企业产值大幅下降，全市经济运行仍保持“稳中有进、进中向好”的良好态势。预计地区生产总值增长8.3%，全社会固定资产投资增长18.7%，社会消费品零售总额增长12.8%，地方公共财政预算收入增长9%，实际利用外资增长31.9%，外贸出口增长12.5%，城镇常住居民人均可支配收入增长10%，农村常住居民人均可支配收入增长12%。市四届人大六次会议确定的主要目标任务基本完成，为实现“十二五”规划的圆满收官和“十三五”顺利开局奠定了坚实基础。

　　五年的艰辛，五年的汗水，五年的收获，五年的辉煌，十堰迈上了新的台阶，跨入了新的阶段，站在了新的起点。成就来之不易，是市委正确领导的结果，是历届市委、市政府“打基础、管长远”成效积累的结果，是市人大、市政协监督帮助的结果，是全市人民共同拼搏的结果，是社会各界参与支持的结果。在此，我谨代表市人民政府，向所有参与和支持十堰发展的同志们、朋友们，表示衷心的感谢、致以崇高的敬意！

　　五年的发展历历在目，积累的经验弥足珍贵。我们深切体会到，推动十堰转型跨越发展，必须坚持发展第一要务，咬定建设区域性中心城市和“两个走在前列”战略目标不放松，坚持做大总量和做优质量两不误；必须坚持改革创新，向改革要活力，向创新要效益；必须坚持绿色发展，大力发展生态经济、绿色产业，实现生态崛起、绿色振兴；必须坚持以人为本，切实增进民生福祉，不断增强人民群众的获得感和幸福感。

　　我们也清醒地认识到，十堰经济社会发展仍然存在不少困难和问题：经济总量不大，质量不高，结构不优，创新不够；城乡发展不平衡，农业基础薄弱，贫困人口较多；少数政府工作人员不作为、慢作为，一些群众反映强烈的问题没有及时解决。对此，我们一定以对人民高度负责的精神，不回避，不懈怠，积极推进解决。 二、“十三五”时期经济社会发展主要目标和任务

　　“十三五”是我市决战贫困、全面建成小康社会的决胜期，也是可以大有作为的发展黄金期。这期间“一带一路”、长江经济带和精准扶贫等国家重大战略加快实施，南水北调移民后扶、水污染防治和水土保持、对口协作等重大政策进一步落地，武十高铁、东风国际合作等重大项目深入推进；同时我市生态环境良好、自然资源富集、工业体系完备、干部作风扎实，我们完全有条件、有能力实现新的跨越。根据《中共十堰市委关于制定十堰市经济和社会发展第十三个五年规划的建议》，我们编制了《十堰市经济和社会发展第十三个五年规划纲要（草案）》（以下简称《纲要草案》），提请大会审议。

　　“十三五”时期全市经济社会发展的指导思想是：高举中国特色社会主义伟大旗帜，全面贯彻党的十八大和十八届三中、四中、五中全会精神，以马克思列宁主义、毛泽东思想、邓小平理论、“三个代表”重要思想、科学发展观为指导，深入贯彻习近平总书记系列重要讲话精神，围绕“四个全面”战略布局，落实创新、协调、绿色、开放、共享发展理念，牢固树立“三维纲要”，坚持生态立市、产业强市、文明兴市，以脱贫攻坚为统领，以生态文明建设为主线，以率先在秦巴山片区全面建成小康社会为目标，外修生态、内修人文，深入推进“一城两带”发展战略，加快构建“一核多支点”发展格局,加快“两个走在前列”进程，建设生态十堰、人文十堰、创新十堰、开放十堰、幸福十堰，不断开创区域性中心城市建设新局面。

　　今后五年经济社会发展的主要奋斗目标是：综合实力和人民生活跨上新台阶，地区生产总值年均增长9%，地方公共财政预算收入年均增长11%，全社会固定资产投资年均增长15%，居民人均可支配收入达到2.2万元，产业迈向中高端水平，城乡发展更趋协调，发展空间格局更加优化，人民生活水平明显提高，贫困人口全部脱贫，贫困村全部出列，贫困县全部摘帽，在秦巴山片区率先脱贫；生态文明建设取得新成效，建成国家生态市和生态文明先行示范区；社会文明程度达到新水平，人民群众精神文化生活更加丰富；改革开放实现新突破，发展环境明显优化，创新创造活力竞相迸发；社会治理开创新局面，实现社会治理制度化和法治化。

　　实现上述目标，我们必须坚决贯彻五大理念引领发展，牢牢抓住精准脱贫统揽发展，善做善成培植支柱产业支撑发展，驰而不息抓好作风建设保障发展。

　　（一）全面推进创新发展，加快培育现代产业体系

　　实施创新驱动战略，坚持以理念创新为先导、科技创新为核心、产业创新为基础、体制机制创新为保障，把十堰建成创新驱动发展的高地。

　　——培育跨越发展新动力。全面深化改革，创优发展环境，全力扩大投资、消费、出口，推动经济发展“保持中高速、迈向中高端”。一是促进投资、消费、出口协调拉动。突出项目投资工作的首位度，构建“规划一批、储备一批、申报一批、建设一批、完成一批”的项目投资梯次推进体系，争取十巫高速、荆安铁路、通用航空等重大基础设施项目启动建设，力争累计完成投资9000亿元；推动消费由生存型向发展型升级、物质型向服务型升级、传统型向新型消费升级，形成新的消费热点；壮大出口企业，培植骨干品牌，培育开放型经济新优势。二是全面激发市场活力。加快转变政府职能，进一步简政放权，全面推行“四个清单”制度和“一枚印章管审批、一个部门管市场、一支队伍管执法”。支持非公有制经济创新发展，实行统一的市场准入制度，加强城乡建设用地、人力资源、金融服务等要素市场建设。全面推行“保姆式”全程服务，建设优良营商环境。三是加强品牌建设。实施质量强市和标准化引领战略，走精品名牌之路，打造一批特色鲜明、竞争力强、市场信誉好的精品名牌，促进十堰产品向十堰品牌提升。

　　——抓住科技创新牛鼻子。深化科技改革，落实“科技十条”，加大知识产权保护和利用，强化各领域关键技术研发和新技术应用，促进重大科技项目成果转化，建设秦巴山片区科技创新中心。着力发现、培养、集聚科技领军人才、企业家人才、高技能人才，建设人才强市。实施“科技企业创业与培育工程”，支持企业创新成长，力争到2020年国家高新技术企业达260家，高新技术产业增加值占GDP的比重达16%。

　　——激发创业创新大能量。完善鼓励创业创新的体制机制，制定和落实优惠政策，降低创业门槛，建立健全创业创新服务体系，大力培育创业创新主体，让大众创业、万众创新蔚然成风，让创业主体从精英小众转为社会大众。切实加强园区建设，加快建设高铁经济区、空港经济区，推进十堰经济技术开发区创新发展，支持县市区园区建设，将全市各级开发区建设成为创业创新的示范平台。

　　——构建现代产业新体系。贯彻《中国制造2025》和“互联网＋”行动计划，坚持效速并进、量质兼取，推动三次产业、信息化与工业化深度融合，带动全产业链转型升级。一是推动汽车产业转型发展。全力服务东风公司，打造世界卡车第一品牌。全面实施地方汽车产业“整车专用化”战略，大力发展新能源商用车，推动汽车产业转型升级、提质增效，努力建设国际商用车之都，汽车工业产值在期末突破2000亿元。二是加快培植旅游、农产品加工、现代服务业三个千亿级产业。优化旅游空间布局，完善旅游产品体系，着力将十堰建设成为“山水城互动、文旅农互融、各要素配套”的国际旅游目的地，旅游综合收入突破1000亿元。推进“四个百万”工程，建设规模基地，开展精深加工，培育特色园区，推进农产品加工标准化、品牌化，产值突破1000亿元。大力发展健康养老、金融保险、信息咨询、文化创意、现代物流等新兴服务业，加快发展电子商务，促进服务业扩规升级，增加值突破1000亿元。三是积极培育战略性新兴产业。以智能化汽车工艺装备，机械手、机器人等为重点，大力发展高端装备制造业，力争产值达到500亿元。突破发展生物医药产业，推进科研攻关，培育龙头企业，力争产值达到300亿元。加强节能减排和循环利用领域重大技术、装备和产品的研发应用，发展生态环保产业，力争产值达到100亿元。大力发展水电、太阳能、风能、生物能等清洁能源，加快高强度钢、新型塑料、复合材料等新材料应用，力争能源产值突破100亿元，新材料产值达到150亿元。“十三五”末，形成以汽车产业为主导，以生态文化旅游、能源、现代服务业、绿色有机农产品加工产业为支撑，以智能装备制造、生物产业、节能环保、新材料产业为重点的“一主四大四新”现代产业体系。

　　（二）全面推进协调发展，深化提升“一核多支点”战略格局

　　紧扣解决发展不平衡、不协调、不可持续问题，切实把调整比例、优化结构作为重大而紧迫的任务，在协调发展中拓宽发展空间，在补齐短板中增强发展后劲。

　　——推动区域协调发展。尊重城市发展规律，统筹空间、规模、产业三大结构，统筹规划、建设、管理三大环节，统筹政府、社会、市民三大主体，提高城市工作的全局性、系统性、可持续性、宜居性和各方推动城市发展的积极性，建设紧凑城市，有效化解各类城市病。推进城区东拓、北扩、西进、南通、中优，完善城市配套功能，加快生态滨江新区建设，推动城市近郊区和辐射区组团发展，加快城市交通、地下综合管网、供（排）水、供气、供电、充电桩、污水和垃圾处理等基础设施建设，建设城市绿地系统，完善城区自然公园、综合公园和社区公园三级公园体系，推行立体绿化，统筹地下空间和地上空间开发利用，市区（含郧阳）建成区面积达150平方公里、人口达150万人。加快竹房城镇带和汉江生态经济带同步协调发展，推动堵河生态经济带建设，大力发展县域经济，切实加强全域综合交通运输网、水利设施网、能源支撑网、智慧城市网建设，完善区域基础设施，支持各县市发挥优势、突出特色、竞相发展，形成“一核”引领、“多点”支撑的发展格局。

　　——推动城乡协调发展。积极推进以人为核心的新型城镇化，构建以城区为核心，县城为支撑，中心镇为节点，一般乡镇和新型农村社区协调发展、互促共进的城镇体系。促进农村劳动力转移就业创业和农民市民化，期末城镇化率达60%。紧扣“绿、净、齐、富、厚、和”建设美丽乡村，大力实施宜居住房、清洁生产、饮水安全、通畅路网、环境治理、乡村绿化等工程建设，发展农村新社区，健全公共服务体系，努力提升农村居民幸福指数。

　　——推动物质文明和精神文明协调发展。狠抓精神文明建设，巩固提升“十星”经验，深化文明城市、文明村镇、文明单位、文明家庭等创建活动，培育和践行社会主义核心价值观，加强思想道德和社会诚信建设，放大“道德群星”示范效应，弘扬中华传统美德，倡导积极向上的生活方式，努力创造团结进取、安定和谐的社会氛围。

　　（三）全面推进绿色发展，打造生态文明样板

　　把生态文明建设贯穿于经济社会发展各方面和全过程，推动形成绿色发展方式和生活方式，协同推进生态美和百姓富。

　　——构建科学合理的空间布局。编制实施《十堰市主体功能区建设方案》，合理确定城镇、农业、生态三类空间比例，划定生态保护红线，生态空间占比高于80%，促进生产空间集约高效、生活空间宜居适度、生态空间山清水秀。

　　——全面节约和高效利用资源。强化约束性指标管理，对能源消耗、建设用地实行总量和强度双控，促进全民节能、节水、节地、节材，全面提高资源利用效率。淘汰落后产能，推进清洁生产，发展循环经济，确保节能减排绩效保持在全省前列。

　　——加大环境治理力度。实施最严格的环保制度，全面开展“蓝天行动”、“清水行动”、“静音行动”、“减废行动”和“净土行动”，统筹推进工业点源、农业面源污染和石漠化、水土流失治理，强化水、大气、土壤、噪音等污染防治，确保全市空气质量优良率达到80%以上，国省市控断面水质达标率达90%以上，城镇生活垃圾无害化处理率达到90%。加快郧阳、郧西、竹溪等水生态文明示范县建设。深入开展“绿满十堰”行动，推进新一轮天然林保护、退耕还林还草，巩固秦巴生物多样性生态功能区地位，确保全市森林覆盖率达到65%以上。

　　——创新生态文明体制机制。编制实施自然资源资产负债表管理模式，健全生态补偿和区域协调机制，让绿色发展的体制机制更加完善。

　　（四）全面推进开放发展，实现内外开放新突破

　　深入实施开放先导战略，坚持内外开放并举、“引进来”和“走出去”并重，不断开创开放发展新局面。

　　——加快建设开放体制。创新管理和服务机制，完善投资贸易机制，营造法治化、便利化的发展环境。加快公路、铁路、航空口岸、电子口岸建设，推进海关、商检平台建设，强化与省内外口岸通关协作，实现“大通关”。建设多层次资本市场，引进武汉股权托管交易中心等要素市场，发展壮大产业投资和创业投资市场，扶持优质企业上市融资和挂牌。

　　——大力推进招商协作。加强产业链招商，积极对接京津冀、长三角、珠三角，实施专业招商和回归工程，大力提升招商质效。创新对口协作机制，推进项目兴办、产业帮扶、市场拓展、劳务输出、人才培养等协作，打造京津冀对口协作示范区。围绕生态农业、文化旅游、工业制造、节能环保、人力资源、科技支持、经贸交流、社会事业等八大领域，确保每年有一批高质量的项目落户十堰。

　　——积极扩大对外开放。对接国家“一带一路”建设，深度融入东风公司全球战略，积极参与广交会等展会活动，纵深拓展国内外两个市场。着力优化出口结构，提升经济外向度，着力培育一批过亿元的外贸出口企业。

　　（五）全面推进共享发展，着力增进民生福祉

　　坚持民生优先、守住底线，扎实推进精准扶贫，切实保障和改善民生，不断提高群众的获得感和幸福感。

　　——坚决打赢扶贫攻坚战。以脱贫攻坚统揽经济社会发展全局，围绕“精准扶贫，不落一人”总要求和“三年集中攻坚、两年巩固提升”总目标，全面实施“36511”精准脱贫计划、“四双”和“结穷亲、帮穷户、拔穷根”精准扶贫工程，强力推进“五个一批”。确保2018年，贫困人口全部脱贫、贫困村全部出列、贫困县（市、区）全部“摘帽”，在秦巴山片区率先实现全面小康。

　　——持续增加城乡居民收入。实施更加积极的就业政策，完善创业扶持政策，促进劳动报酬合理增长，增加居民财产性收入；赋予农民更多财产权利，拓展非农增收渠道，多措并举，努力做到城乡居民收入增长高于经济增长。

　　——不断完善公共服务供给。坚持普惠性、保基本、均等化、可持续方向，提高公共服务供给能力。继续扩大社会保险覆盖面，实施全民参保计划，不断提高统筹层次和保障水平。健全覆盖城乡的社会保障体系，完善以扶老、助残、爱幼、济困为重点的社会福利制度，加快构建医养结合的养老服务体系。做好教育工作，发展普惠学前教育，均衡推进义务教育，普及高中教育，强化职业教育，办好特殊教育，支持湖北医药学院、湖北汽车工业学院、郧阳师专、湖北工业职业技术学院按各自目标加快建设发展；建设“健康十堰”，加大传染病防控力度，实现基本医疗卫生制度全覆盖，完善医疗服务体系，建设名医、名科、名院，打造特色医疗品牌，建成区域性医疗中心；全面落实计生政策，做好计生服务；促进文体新广事业全面发展，深入挖掘、保护和传承地方传统文化，推进全民健身运动，建成公共文化服务体系和现代文化市场管理体系。规范房地产开发秩序，加大保障性安居工程建设力度，提高住房保障能力。

　　——切实加强民主法治建设。积极推进政务信息公开，完善重大决策民意听证、专家论证、政府会商程序，强化行政监察和社会监督，推进行政决策民主化。全面依法治市，推进依法行政。行使好立法提案权，充分发挥政府在立法工作中的基础和支撑作用，依法制定政府规章，提高法治建设水平。完善公共法律服务体系，大力建设法治十堰。加强平安十堰建设，扎实做好信访、安全生产和食品药品安全工作。重视国防动员，支持国防建设。

　　三、扎实做好2016年工作

　　2016年是“十三五”规划的开局之年，是全面建成小康社会决胜阶段的首战之年，做好今年的工作意义深远、责任重大。面对复杂的发展形势和经济下行持续加大的压力，我们将趋利避害，扬长补短，确保开好局、起好步。

　　今年经济社会发展的主要预期目标是：地区生产总值增长9%；规模以上工业增加值增长10%；全社会固定资产投资增长18%；社会消费品零售总额增长13%；地方公共财政预算收入增长10%；外贸出口增长10%；城乡居民收入高于经济增长水平；城镇登记失业率控制在4.5%以内；居民消费价格指数和节能减排控制在省定目标以内。

　　（一）聚焦聚力精准脱贫

　　集中精力扶贫攻坚，锁定建档立卡贫困人口，创新扶贫举措，强化要素投入，实施挂图作战，严格责任考核，完善奖惩机制，确保减少贫困人口16万人，“四双”驻村帮扶村30%基本脱贫，85个重点贫困村出列。

　　建立完善攻坚作战、政策支撑、精准管理、攻坚责任四大体系，打好易地扶贫搬迁、特色产业开发、基础设施建设、转移培训就业、社会保障和公共服务五大硬仗，创新精准识别、驻村帮扶、片区攻坚、生态扶贫、资金整合、贫困村户退出六大机制，实施182个村整村推进，新建改造特色基地10万亩，培育示范基地1000个，启动建设旅游扶贫村40个，建设电商服务示范点100个。完成扶贫搬迁2万户8万人。组织“雨露计划”等技能培训1万人，贫困人口转移就业5000人以上。实施保障兜底扶贫工程，加大对贫困户医疗救助力度，降低大病保险起付线，提高就医和大病保险报销比例；落实“双线合一”政策，把丧失劳动能力的农村贫困人口，全部纳入“低保”和“五保”范围。

　　（二）强力扩大投资和消费需求

　　——狠抓项目投资。以大项目推动调结构，以大投入促进稳增长，确保全社会固定资产投资达到1500亿元以上。锲而不舍争取国家投资。吃透政策，跟踪信息，紧密衔接，争取一批打基础、补短板、增后劲、惠民生的投资项目。千方百计撬动社会投资。大力创新投融资机制，推行PPP模式，密切与央企合作。充分利用产业基金、国债等资金，着力激活和吸引民间投资；创优信用环境，改善金融服务，力争全年新增贷款200亿元。全力以赴促进投资落地。突出抓好15个省级和127个市级重点项目建设，加快推进武十高铁、京能热电、夹河关水电站、孤山水电站建设进度，开工建设238省道竹溪至竹山段、279省道河南至丹江口段、丹江口至房县段、283省道房县九道至义渡段等公路项目和郧西县兰滩口大桥，确保丹江口库区生态旅游公路、十竹公路建成通车；做好十堰至陕西西安高铁、十巫、十淅高速等项目前期工作；抓好城市综合管廊、热力管网、十堰火车站北广场、十竹天然气管线等重大项目建设。

　　——着力推动消费升级。大力发展服务消费、信息消费、绿色消费、时尚消费、品质消费、农村消费，重点推进旅游休闲、健康养老、家政服务、教育培训、电子信息、文体娱乐等消费。完善商品市场网络，加快“批发出城、零售进城”步伐，推动农村流通网络升级和城市商圈服务功能提升。加强质量监管，规范市场秩序，多措并举释放消费潜力。

　　——全面促进创业创新。发展众创空间，推动创新与创业结合、线上与线下结合、孵化与投资结合，确保大众创业万众创新取得积极成效。加快中国（十堰）商用车研究院、院士工作站、技术交易中心等创新创业平台建设，着力提高科技成果的转化率和产业化率，大力推进电商创业，培育壮大市场主体。新认定高新技术企业26家，高新技术产业增加值增长10 %以上。

　　——不断壮大实体经济。推进供给侧结构性改革，大力促进去产能、去库存、去杠杆、降成本、补短板。积极落实结构性减税、电价调整、降低社保费等优惠政策，降低企业生产成本。加强政银企对接，产业链上下游对接、产学研对接和劳动力培训用工对接，缓解企业融资、技术、用工等方面的难题。坚持领导联系服务重点企业制度，落实绿色通道、包保服务等工作机制，扩大“过桥基金”规模，帮助企业解决具体困难，把更多的政府资源投入到服务实体经济发展中去。

　　（三）大力促进一二三产业健康发展

　　——全面促进工业结构升级优化。坚持扩总量、提质量、优结构、增效益并重，推进工业化、信息化融合发展，着力形成一主多元、多极支撑的工业发展格局。一是强力推进汽车产业升级增效。全力服务东风公司，强化东风商用车公司在国内国际市场的领先地位。推动动力总成等重大项目建设。支持东风特商、东风装备、东风零部件、东风小康、三环加快发展。扎实推进“整车专用化”战略，支持发展高端、重型专用车，培育大运、驰田、海龙等地方专用车品牌，力争专用车事业有新突破。二是积极发展新兴产业。大力培育新能源汽车、高端装备制造、电子信息、新型建材、生物医药等新兴产业。扶持新能源汽车及富士康、中广核、骐通机器人、佳恒公司轨道交通等重大项目建设，加快推进生物产业园区建设，争取国药控股项目投产见效。三是培育壮大骨干企业。建好用好产业引导基金，深入实施中小企业成长工程，推进企业兼并重组，促进中小企业扩能进规，培育壮大一批骨干企业，确保全年新增规上企业100家以上。

　　——大力发展高效生态农业。用发展新理念破解“三农”工作新难题，推动农业持续增效、农民稳定增收。一是夯实现代农业基础，提高农业质量效益。着力构建农业产业体系、生产体系、经营体系，推动粮经饲统筹、农林牧结合、种养加一体、一二三产业融合发展。大规模推进高标准农田和农田水利建设，优化农业生产结构和区域布局，加快培育新兴农民。新（改）建特色基地28万亩，新建县乡道路100公里、通村水泥路1000公里，除险加固水库50座，解决农村安全饮水13.5万人，治理中小河流4条，水土保持、生态修复200平方公里。二是推进农村产业融合，促进农民收入较快增长。促进农产品粗加工、精深加工及其综合利用加工协调发展，提高农产品加工转化率和附加值，增加对农民增收的带动能力。新增重点龙头企业和规上农产品加工企业30家以上，农产品加工产值达420亿元以上。发展多种形式的适度经营，新增农民专业合作社100家、家庭农场（牧场）150家、专业大户1000家。

　　——加快现代服务业提档升级。坚持生产性服务业和生活性服务业并重、现代服务业与传统服务业并举，推动服务业优质高效发展。一是加速发展生态文化旅游业。大力拓展旅游业态，完善旅游链条，培植精品线路，升级旅游产品，扩大武当山、太极湖及城郊生态游憩带等重点旅游线路的影响力和吸引力，力争年实现旅游接待4700万人次，年综合收入360亿元。二是加快发展商贸物流业。加速推进万达、华西农商城，昌升、林安、亨运物流，寿康华悦城等大型项目建设，启动华彬健康产业、绿城综合体建设，推进空港经济圈、高铁站经济圈、京能铁路物流圈、农村物流融合发展试验区的规划建设。三是大力发展新兴服务业。重点加快电子商务、健康养老、金融服务、文化体育、信息服务等产业发展，实施电子商务进农村、综合服务进社区、特色农产品流通升级增效三大工程。鼓励社会力量兴办养老机构，加快城乡社区居家养老服务站和环丹江口库区生态旅游养生养老项目建设。

　　（四）深入推进重点领域改革和对外开放

　　——持续推进改革攻坚。深化行政审批制度改革，实施政务服务标准化示范创建，健全市、县、乡、村四级政务服务体系，完成“三个一”改革试点。深化经济领域改革，健全公开规范的土地流转市场，推进土地矿产供应和交易机制改革，启动不动产统一登记,完成农村宅基地、集体建设用地确权登记发证工作。推进检验检测机构改革，建设社会化公共技术检测平台。深化城乡领域改革，改革户籍制度，放宽户口迁移政策。深化民生领域改革，推进机关事业单位养老保险、工伤保险和公立医院改革，落实降低社会保险费“五险一金”精简归并措施，抓好社会化养老服务综合改革试点工作。深化生态文明体制改革。促进司法体制改革。

　　——强力开展招商引资。越是经济下行压力大越要加大招商力度。要精准对接，定向发力，加强在谈、签约项目跟踪服务，着力提高招商实效。力争国寿养生、比克电池、沃特玛电池、国药医药等项目取得实质进展，华彬功能饮料、富士康与中广核新能源、鄂西北药材电商批发城和中国义乌展贸城等项目开工建设，天喜达日化、正发电子、葵花药业等项目建成投产，确保全年引进新项目100个以上。

　　——努力扩大对外开放。筹建十堰电子口岸，引导企业开拓海外市场，扩大汽车及零部件、香菇、木耳等优势特色产品出口规模。鼓励和支持企业积极参与对外合作。加强对口协作，进一步拓展协作领域，遴选并实施好年度协作项目，加快推进十堰中关村基地建设。

　　（五）科学统筹区域发展

　　——着力提升城区服务功能。加快市政道路和绿道建设，新（改）建10条、续建15条主次干道，建成十堰大道、机场路绿道和一批市内山体公园、街头绿地。完成黄龙镇、柏林镇供水管网工程，新增天然气入户2万户以上。加强数字化城管建设，推进城市管理规范化、标准化。开辟6条以上航线。

　　——高效发展县域经济。扶持郧阳区农产品加工、丹江口市水产业开发、郧西新能源、竹溪光电子、竹山绿色食品、房县生物医药等重点产业发展，不断壮大县域综合实力，力争县域GDP增长10%以上，县域工业增加值增长12%以上。

　　——提高新农村建设水平。抢抓竹房城镇带建设时间延长、范围扩大和汉江生态经济带列为省级战略的机遇，集中打造一批特色城镇。继续推进郧阳区茶店镇、竹山县宝丰镇示范乡镇和25个国家级重点镇，省中心镇、特色镇建设，建好一批农村新社区示范片区，切实提升城镇综合承载力。加快西沟、茅塔生态旅游区等10个城区农村重点片区建设。

　　（六）切实加强生态文明建设

　　——大力加强生态建设。持续开展“清水行动”，完成“五河”综合整治，推进国家良好湖泊生态保护试点项目建设。实现污水处理厂委托运营管理全覆盖。加大植树造林、生态修复力度，建设环库周公路生态景观林。加快创建国家生态文明先行示范区，扎实推进“五城联创”，确保全国城市文明指数测评取得更好成绩、国家环保模范城市基本达标，争取国家森林城市创建通过考核验收。完成农村环境综合整治600个村，支持丹江口、竹溪建成省级生态县（市），支持竹溪申报国家公园试点。

　　——深入推进环境保护。继续淘汰落后产能，加大节能减排力度。加强地质环境保护，科学防治地灾。加快推进垃圾转运站建设，完善生活垃圾转运收集和无害化处理设施。加强扬尘控制和噪声防治，全面实施“大气十条”和“水十条”，力争全市空气质量优良率达到80%。

　　（七）着力保障和改善民生

　　——大力发展普惠民生。多渠道开发就业岗位，实现城镇新增就业3.5万人，转移农村劳动力3万人。进一步扩大社会保险覆盖面。提高城乡低保、农村“五保”保障标准。加快推进市养老护理院、精神病人福利院和郧阳区社会福利中心等项目建设。全面做好移民后扶工作，改造老水库避险解困危房5000户。加大棚户区和老旧小区改造力度，改造农村危房1.5万户，开工保障房4.7万套。

　　——全面发展社会事业。扩大城镇普惠性幼教资源，加快中小学校标准化建设，深入推进“联校协作发展”，均衡发展义务教育，加快城区4所高中、职中和全市8所思源学校建设，大力支持职业教育、高等教育内涵发展。深化公立医院综合改革，建立分级诊疗制度，巩固完善基本药物制度，支持社会资本办医，实施全面两孩政策。推动公共文化服务体系均等化发展，加强地方文化挖掘保护，启动地面数字电视工程，广泛开展全民健身运动，开工建设市奥林匹克健身公园。

　　——不断创新社会治理。推进城乡网格化管理，健全立体化社会治安防控体系，实施“诚信工程”，建设“平安十堰”、“信用十堰”。坚持开展矛盾隐患大排查大调处活动，积极预防和妥善处置群体性事件和突发公共安全事件。全面落实安全生产“一岗双责”，坚决遏制重特大安全事故发生。切实维护食品药品和特种设备安全。

　　（八）努力建设人民满意政府

　　各级政府及其工作人员必须坚持正确政治方向，自觉践行“三严三实”要求，勤勉敬业，善做善成，不负人民重托，赢得群众信任。

　　——明责担责尽责。强化责任意识，视责如山，守责如城，履责如冰，担好深化改革之责，履好推动发展之责，负好服务民众之责，做到为人无愧于己、为政无愧于民、忠诚无愧于党。强化法治意识，法定职责必须为、法无授权不可为，坚决执行人大及其常委会的决议决定，自觉接受其法律监督、工作监督和政协民主监督，认真办理代表、委员的建议和提案，广泛听取社会各界意见建议，推进依法、科学、民主决策。强化担当意识，以身许党许国，以身报党报国，迎困难上、向矛盾行、在险处为；敢于动真碰硬，耻于八面玲珑；该放手时决不事无巨细，需上阵时定要亲力亲为。强化实干意识，功成不必在我但功成定必有我，求真务实、狠抓落实，不计名利、不图虚功，多谋打基础、添后劲、利长远的实举，多办顺民意、惠民生、得民心的实事，多出经得起实践、人民、历史检验的实绩。

　　——提气提神提能。提振士气、升腾气场，始终保持昂扬向上的朝气、拼搏进取的锐气、干事创业的豪气，集聚裂变升华的巨大能量，形成人心思齐、人心思进、人心思干、人心思成的浓厚氛围。振奋精神、凝神聚力，始终保持竞进有为、争先创优、决战决胜的姿态，自我加压、自我突破、负重前行，全面提升精神区位。提高能力、增长才干，始终保持与时俱进、锐意改革、开拓创新的风貌，切实补齐“知识缺失、能力不足、本领恐慌”的短板，勤于学、敏于思、精于业，向群众讨方法、向先进学经验，向实践找答案，获取“活知识”，学到“真功夫”，掌握“硬本领”，在学、思、践、悟中不断提升驾驭市场经济能力、应对复杂局面能力、依法行政能力、统筹协调能力。

　　——清风清境清政。始终坚持“永远在路上”、“现在进行时”，驰而不息改进作风，持之以恒倡简戒奢，大兴清简务实之风，以清明的政风带动民风社风，培育风清气正的社会环境。坚持以群众满意、社会认可为目标，提供“零距离”服务，实现“无缝式”对接，敬民、爱民、抚民、富民，亲商、安商、扶商、兴商，打造开明开放的营商环境。转变政府职能，提升行政效能，放活市场“无形之手”，完善社会“自治之手”，用好政府“有形之手”，该管的管到位、该放的放彻底，不越位、不缺位、不错位、做到位，营造顺畅高效的政务环境。

　　——治庸治懒治腐。坚持从严治政，既向贪污腐化“显腐败”下手，又对庸官懒政“隐腐败”问责，坚决治理庸官懒政和腐败行为，让庸官无地自容，让懒官无所遁形，让贪官无处藏身。强化效能评价，严格绩效考核，重手问效治庸，突出解决干部能力弱、绩效差，思想疲、效率低，不创新、不谋事，不会作为等“平庸”问题；严厉问责治懒，强化责任追究，坚决杜绝有劲不使、有力不用，遇事推诿、遇难绕道，不求有功、但求无过，为官不为、得过且过等懒政惰政怠政现象；高压问廉治腐，坚持把纪律挺在前面，严守《准则》《条例》，落实主体责任，履行“一岗双责”，以零容忍的态度坚决查处各类违规违纪行为，锻造清廉从政、事业有为的干部队伍和公务员队伍。

　　各位代表！今年将继续办好十件实事：一是新建通村水泥路1000公里；二是建设标准化贫困村卫生室100个；三是完成城区棚户区改造及综合整治城区老旧小区7000户；四是建设城区普惠性幼儿园10所；五是建好市特殊教育学校；六是建设社区居家养老服务中心60个；七是新建城区街头、社区、山体游园10个；八是完成天然气对接入户2万户；九是改造城区旱厕20座；十是完成就业创业培训3万人，扶持创业5000人，创业带动就业2万人。

　　各位代表，实现宏伟蓝图，成就兴市伟业，惟赖躬身实干，切需勠力同心。让我们在市委的坚强领导下，乘风破浪，加油奋发，克难猛进，共同开创生态文明建设和转型跨越发展走在全省前列、区域性中心城市建设的崭新局面！
